# Supplementary material for: Identification of Volatile Markers during Early Zygosaccharomyces rouxii Contamination in Mature and Immature Jujube Honey
Source: Foods. 2023 Jul 18;12(14):2730. doi: 10.3390/foods12142730 (PMC10379421; doi:10.3390/foods12142730)
Supplement: Supplementary file 1 [file foods-12-02730-s001.zip › Supplementary Tables.pdf]

Supplementary table 1 Change of relative peak area of volatile compounds in HMH during storge (mean±SD) and correlation analysis with storage 3-30 days.

Metabolite	ck	3d	7d	15d	30d	Coefficient of correlation(r)
Butanoic acid, methyl ester	0.00±0.00	0.00±0.00	2.00±0.08[a]	2.03±0.06[a]	2.03±0.11[a]	0.869**
3-Pentanone, 2-methyl-	0.00±0.00	2.00±0.09[b]	2.00±0.10[b]	2.00±0.05[b]	2.33±0.03[a]	0.780**
Toluene	2.00±0.00[a]	2.00±0.00[a]	2.00±0.01[a]	1.99±0.01[a]	2.00±0.01[a]	-0.087
Octane	2.00±0.10[b]	2.21±0.07[a]	2.13±0.01[a]	2.17±0.01[a]	2.00±0.05[b]	-0.055
p-Xylene	2.00±0.01[a]	2.00±0.01[a]	2.00±0.09[a]	2.05±0.02[a]	2.03±0.01[a]	0.343
Tetrahydrofuran, 2-methyl-5-pentyl-	2.00±0.10[c]	2.00±0.07[c]	1.97±0.06[c]	2.41±0.01[a]	2.13±0.03[b]	0.557*
o-Xylene	2.00±0.06[a]	2.00±0.10[a]	2.00±0.05[a]	2.00±0.07[a]	2.00±0.06[a]	0.000
Nonane	1.90±0.07[b]	2.00±0.07[b]	2.00±0.05[b]	2.51±0.04[a]	1.97±0.07[b]	0.403
Pentanoic acid, 3-methyl-, methyl ester	0.00±0.00	0.00±0.00	2.00±0.09[a]	2.03±0.04[a]	2.00±0.08[a]	0.865**
2-Furanmethanol, 5-ethenyltetrahydro-α,α,5-trimethyl-, cis-	2.07±0.00[a]	2.02±0.00[b]	2.06±0.00[a]	2.00±0.01[c]	2.00±0.01[c]	-0.746**
α-Methyl-α-[4-methyl-3-pentenyl]oxiranemethanol	2.04±0.05[b]	2.51±0.06[a]	2.00±0.08[b]	2.00±0.03[b]	2.00±0.05[b]	-0.401
Linalool	0.00±0.00	2.00±0.08[b]	2.17±0.01[a]	2.00±0.06[b]	2.00±0.05[b]	0.689**
Nonanal	2.00±0.01[c]	2.00±0.01[c]	2.07±0.00[a]	2.02±0.00[b]	2.00±0.00[c]	0.107
Decanal	2.00±0.01[c]	2.00±0.00[c]	2.00±0.01[c]	2.05±0.00[b]	2.18±0.00[a]	0.839**
Nonanoic acid, methyl ester	2.03±0.00[d]	2.04±0.00[c]	2.04±0.00[b]	2.05±0.00[a]	2.05±0.00[a]	0.936**
2-Nonenoic acid, methyl ester	0.00±0.00	2.00±0.05[a]	2.03±0.06[a]	1.97±0.08[a]	2.00±0.01[a]	0.700**

Undecanal	0.00±0.00	2.00±0.05[a]	1.95±0.10[a]	2.00±0.06[a]	0.00±0.00	0.000
Undecanoic acid, 10-methyl-, methyl ester	1.98±0.00[e]	2.00±0.00[d]	2.03±0.00[b]	2.04±0.00[a]	2.01±0.00[c]	0.672**
Cedrol	0.00±0.00	0.00±0.00	2.06±0.04[a]	1.97±0.06[a]	2.00±0.09[a]	0.856**
Hexadecanoic acid, methyl ester	2.02±0.00[b]	2.00±0.00[c]	2.05±0.00[a]	2.00±0.00[c]	2.00±0.00[c]	-0.344
13-Octadecenoic acid, methyl ester	0.00±0.00	0.00±0.00	0.00±0.00	2.00±0.05[a]	2.00±0.12[a]	0.865**

Denotes: ck: The blank group of no *Zygosaccharomyces rouxii* was added; **: at the 0.01 level (two-tailed), the correlation is significant; *: at the 0.05 level (two-tailed), the correlation is significant.

Supplementary table 2 Change of relative peak area of volatile compounds in LMH during storge (mean±SD) and correlation analysis with storage 3-30 days.

Metabolite	ck	3d	7d	15d	30d	Coefficient of correlation(r)
Butanoic acid, methyl ester	0.00±0.00	0.00±0.00	0.00±0.00	0.00±0.00	3.94±0.00^{a}	0.707**
3-Pentanone, 2-methyl-	0.00±0.00	0.00±0.00	0.00±0.00	0.00±0.00	2.00±0.34^{a}	0.699**
Toluene	2.00±0.00^{d}	2.00±0.01^{d}	2.06±0.00^{b}	2.01±0.00^{c}	2.07±0.00^{a}	0.684**
Octane	2.03±0.06^{c}	2.00±0.10^{c}	2.00±0.05^{c}	2.20±0.00^{b}	2.90±0.05^{a}	0.787**
p-Xylene	2.00±0.06^{a}	2.00±0.07^{a}	2.00±0.02^{a}	2.00±0.08^{a}	2.00±0.01^{a}	0.000
Tetrahydrofuran, 2-methyl-5-pentyl-	0.00±0.00	0.00±0.00	0.00±0.00	0.00±0.00	2.14±0.03^{a}	0.707**
o-Xylene	2.00±0.14^{c}	2.00±0.05^{c}	2.00±0.10^{c}	2.51±0.02^{b}	12.30±0.05^{a}	0.733**
Nonane	2.31±0.04^{a}	2.00±0.05^{b}	2.00±0.06^{b}	2.00±0.05^{b}	2.30±0.05^{a}	-0.022
Pentanoic acid, 3-methyl-, methyl ester	0.00±0.00	0.00±0.00	0.00±0.00	0.00±0.00	2.51±0.02^{a}	0.707**
2-Octenal, (E)-	0.00±0.00	2.00±0.13^{a}	2.00±0.05^{a}	2.02±0.06^{a}	2.00±0.07^{a}	0.707**
2-Furanmethanol, 5-ethenyltetrahydro-α,α,5-trimethyl-, cis-	2.00±0.00^{b}	2.00±0.01^{b}	2.06±0.00^{a}	2.00±0.01^{b}	2.00±0.01^{b}	-0.021
Linalool	0.00±0.00	0.00±0.00	0.00±0.00	0.00±0.00	2.00±0.07^{a}	0.707**
Nonanal	2.00±0.01^{b}	2.00±0.00^{b}	2.07±0.00^{a}	2.00±0.01^{b}	2.00±0.01^{b}	-0.031
Decanal	1.99±0.01^{c}	2.00±0.00^{b}	2.00±0.00^{b}	2.00±0.01^{b}	2.14±0.00^{a}	0.725**
Nonanoic acid, methyl ester	2.03±0.00^{e}	2.03±0.00^{d}	2.04±0.00^{a}	2.04±0.00^{b}	2.04±0.00^{c}	0.541*
2-Nonenoic acid, methyl ester	0.00±0.00	2.00±0.05^{b}	2.39±0.03^{a}	2.00±0.10^{b}	2.00±0.07^{b}	0.622**

Undecanal	0.00±0.00	2.00±0.08[a]	2.00±0.05[a]	2.00±0.06[a]	0.00±0.00	0.000
Undecanoic acid, 10-methyl-, methyl ester	1.97±0.00[e]	2.03±0.00[c]	2.04±0.00[b]	2.14±0.00[a]	2.02±0.00[d]	0.522*
Cedrol	0.00±0.00	0.00±0.00	2.00±0.09[a]	2.00±0.02[a]	0.00±0.00	0.289
Hexadecanoic acid, methyl ester	2.00±0.01[c]	2.00±0.00[c]	2.00±0.01[c]	2.01±0.01[b]	2.04±0.00[a]	0.739**

Denotes: ck: The blank group of no *Zygosaccharomyces rouxii* was added; **: at the 0.01 level (two-tailed), the correlation is significant; *: at the 0.05 level (two-tailed), the correlation is significant.

Supplementary table 3 Change of relative peak area of volatile compounds in HIH during storge (mean±SD) and correlation analysis with storage 3-30 days.

Metabolite	ck	3d	7d	15d	30d	Coefficient of correlation(r)
Butanoic acid, methyl ester	0.00±0.00	0.00±0.00	0.00±0.00	2.03±0.00[a]	0.00±0.00	0.354
3-Pentanone, 2-methyl-	0.00±0.00	0.00±0.00	2.00±0.03[b]	2.00±0.06[b]	3.24±0.03[a]	0.974**
Toluene	2.00±0.01[b]	2.00±0.01[b]	2.00±0.01[b]	2.00±0.01[b]	2.06±0.00[a]	0.706**
Octane	2.09±0.00[b]	2.00±0.01[c]	2.00±0.01[c]	2.08±0.00[b]	2.10±0.00[a]	0.331
p-Xylene	1.91±0.00[d]	2.00±0.03[c]	2.00±0.07[c]	2.37±0.02[b]	2.19±0.01[b]	0.779**
Tetrahydrofuran, 2-methyl-5-pentyl-	2.00±0.01[b]	1.99±0.01[b]	2.00±0.01[b]	2.06±0.00[a]	2.00±0.00[b]	0.371
o-Xylene	2.00±0.05[c]	2.00±0.04[c]	2.00±0.04[c]	2.33±0.06[a]	2.19±0.00[b]	0.726**
Nonane	2.00±0.08[b]	2.03±0.01[b]	2.58±0.01[a]	2.54±0.03[a]	2.00±0.03[b]	0.266
Heptanal	2.00±0.08[c]	2.00±0.09[c]	2.58±0.01[b]	3.89±0.05[a]	2.00±0.02[c]	0.364
Pentanoic acid, 3-methyl-, methyl ester	2.07±0.00[b]	2.00±0.01[d]	2.00±0.01[d]	2.10±0.00[a]	2.04±0.01[c]	0.164
Heptanoic acid, methyl ester	2.00±0.06[a]	2.00±0.03[a]	0.00±0.00	0.00±0.00	0.00±0.00	-0.866**
2-Furanmethanol, 5-ethenyltetrahydro-α,α,5-trimethyl-, cis-	2.01±0.00[b]	2.00±0.01[b]	2.00±0.01[b]	2.00±0.01[b]	2.05±0.00[a]	0.597*
α-Methyl-α-[4-methyl-3-pentenyl]oxiranemethanol	2.00±0.07[a]	2.00±0.09[a]	2.00±0.06[a]	2.00±0.15[a]	2.00±0.04[a]	0.000
Linalool	2.00±0.01[c]	2.04±0.00[b]	2.09±0.00[a]	2.00±0.01[c]	2.00±0.00[c]	-0.124
Nonanal	2.07±0.00[a]	2.00±0.01[b]	2.00±0.01[b]	2.07±0.00[a]	2.00±0.01[b]	-0.250
Decanal	2.02±0.00[c]	2.00±0.01[d]	2.00±0.00[d]	2.03±0.00[b]	2.05±0.00[a]	0.612*

Nonanoic acid, methyl ester	2.07±0.00^{a}	2.07±0.00^{a}	2.07±0.01^{a}	2.03±0.00^{b}	2.03±0.02^{b}	-0.858**
2-Nonenoic acid, methyl ester	0.00±0.00	2.36±0.03^{a}	2.00±0.07^{c}	2.24±0.02^{b}	1.97±0.07^{c}	0.621*
Undecanal	0.00±0.00	2.00±0.06^{a}	2.00±0.08^{a}	0.00±0.00	2.00±0.00	-0.288
Tetradecane, 2,6,10-trimethyl-	2.00±0.03^{a}	2.00±0.07^{a}	0.00±0.00	0.00±0.00	0.00±0.00	-0.866**
Undecanoic acid, 10-methyl-, methyl ester	1.99±0.00^{c}	2.00±0.01^{b}	2.00±0.01^{b}	2.00±0.00^{b}	2.03±0.01^{a}	0.815**
Cedrol	2.00±0.06^{a}	2.00±0.12^{a}	2.00±0.06^{a}	2.00±0.08^{a}	2.00±0.02^{a}	0.146
Hexadecanoic acid, methyl ester	2.00±0.08^{b}	2.19±0.00^{a}	2.00±0.00^{b}	1.97±0.08^{b}	1.97±0.05^{b}	-0.431
13-Octadecenoic acid, methyl ester	0.00±0.00	0.00±0.00	0.00±0.00	0.00±0.00	1.81±0.00^{a}	0.707**

Denotes: ck: The blank group of no *Zygosaccharomyces rouxii* was added; **: at the 0.01 level (two-tailed), the correlation is significant; *: at the 0.05 level (two-tailed), the correlation is significant.

Supplementary table 4 Change of relative peak area of volatile compounds in LIH during storge (mean±SD) and correlation analysis with storage 3-30 days.

Metabolite	ck	3d	7d	15d	30d	Coefficient of correlation(r)
Butanoic acid, methyl ester	0.00±0.00	0.00±0.00	0.00±0.00	0.00±0.00	2.00±0.01[a]	0.707**
3-Pentanone, 2-methyl-	0.00±0.00	2.00±0.05[b]	2.42±0.01[a]	2.00±0.05[b]	1.90±0.16[b]	0.629**
Toluene	2.04±0.02[c]	2.00±0.01[d]	2.07±0.00[b]	2.09±0.00[a]	2.00±0.00[d]	0.035
Octane	2.00±0.01[b]	2.00±0.01[b]	2.05±0.00[a]	2.07±0.00[a]	2.00±0.01[b]	0.332
p-Xylene	2.00±0.00[c]	2.00±0.05[c]	2.41±0.05[a]	2.13±0.06[b]	2.00±0.00[c]	0.115
Tetrahydrofuran, 2-methyl-5-pentyl-	2.00±0.01[d]	2.03±0.00[c]	2.06±0.00[a]	2.04±0.00[b]	2.00±0.01[d]	0.069
o-Xylene	2.00±0.08[b]	2.00±0.09[b]	2.39±0.03[a]	2.39±0.01[a]	2.00±0.03[b]	0.279
Nonane	2.38±0.07[a]	2.00±0.05[d]	2.48±0.08[a]	2.29±0.06[b]	2.23±0.07[c]	-0.012
Heptanal	0.00±0.00	0.00±0.00	2.27±0.02[b]	2.30±0.02[a]	0.00±0.00	0.290
Pentanoic acid, 3-methyl-, methyl ester	0.00±0.00	2.00±0.01[c]	2.03±0.00[b]	2.08±0.01[a]	2.00±0.00[c]	0.713**
Heptanoic acid, methyl ester	2.00±0.19[a]	1.70±0.05[b]	0.00±0.00	0.00±0.00	0.00±0.00	-0.882**
2-Octenal, (E)-	0.00±0.00	2.00±0.09[c]	2.46±0.03[b]	3.35±0.02[a]	2.00±0.07[c]	0.689**
2-Furanmethanol, 5-ethenyltetrahydro-α,α,5-trimethyl-, cis-	1.99±0.00[b]	1.99±0.00[c]	2.03±0.00[a]	2.03±0.00[a]	2.00±0.01[b]	0.387
Linalool	1.99±0.01[b]	1.96±0.01[c]	2.00±0.01[b]	2.08±0.00[a]	2.00±0.01[b]	0.497
Nonanal	2.00±0.01[c]	1.97±0.00[d]	2.08±0.00[a]	2.08±0.00[b]	1.94±0.00[e]	-0.042
Decanal	2.00±0.01[b]	2.00±0.00[b]	1.97±0.00[c]	2.06±0.00[a]	2.00±0.00[b]	0.282

Nonanoic acid, methyl ester	2.00±0.01[b]	2.00±0.01[b]	2.08±0.00[a]	2.09±0.00[a]	2.00±0.01[b]	0.301
2-Nonenoic acid, methyl ester	0.00±0.00	2.00±0.05[c]	2.37±0.00[b]	2.66±0.03[a]	2.00±0.09[c]	0.703**
Undecanal	0.00±0.00	0.00±0.00	2.96±0.02[a]	2.00±0.07[b]	0.00±0.00	0.226
Tetradecane, 2,6,10-trimethyl-	2.00±0.09[a]	0.00±0.00	0.00±0.00	0.00±0.00	0.00±0.00	-0.706**
Undecanoic acid, 10-methyl-, methyl ester	2.00±0.01[b]	2.00±0.01[b]	2.02±0.00[a]	1.99±0.00[b]	2.00±0.00[b]	-0.113
Cedrol	2.00±0.12[a]	0.00±0.00	0.00±0.00	0.00±0.00	0.00±0.00	-0.706**
Hexadecanoic acid, methyl ester	2.00±0.06[a]	2.00±0.00[a]	2.00±0.07[a]	2.00±0.10[a]	2.03±0.06[a]	0.173

Denotes: ck: The blank group of no *Zygosaccharomyces rouxii* was added; **: at the 0.01 level (two-tailed), the correlation is significant; *: at the 0.05 level (two-tailed), the correlation is significant.
