# Supplementary figures and images for: Identification of Volatile Markers during Early Zygosaccharomyces rouxii Contamination in Mature and Immature Jujube Honey
Source: Foods. 2023 Jul 18;12(14):2730. doi: 10.3390/foods12142730 (PMC10379421; doi:10.3390/foods12142730)

## Slide 1
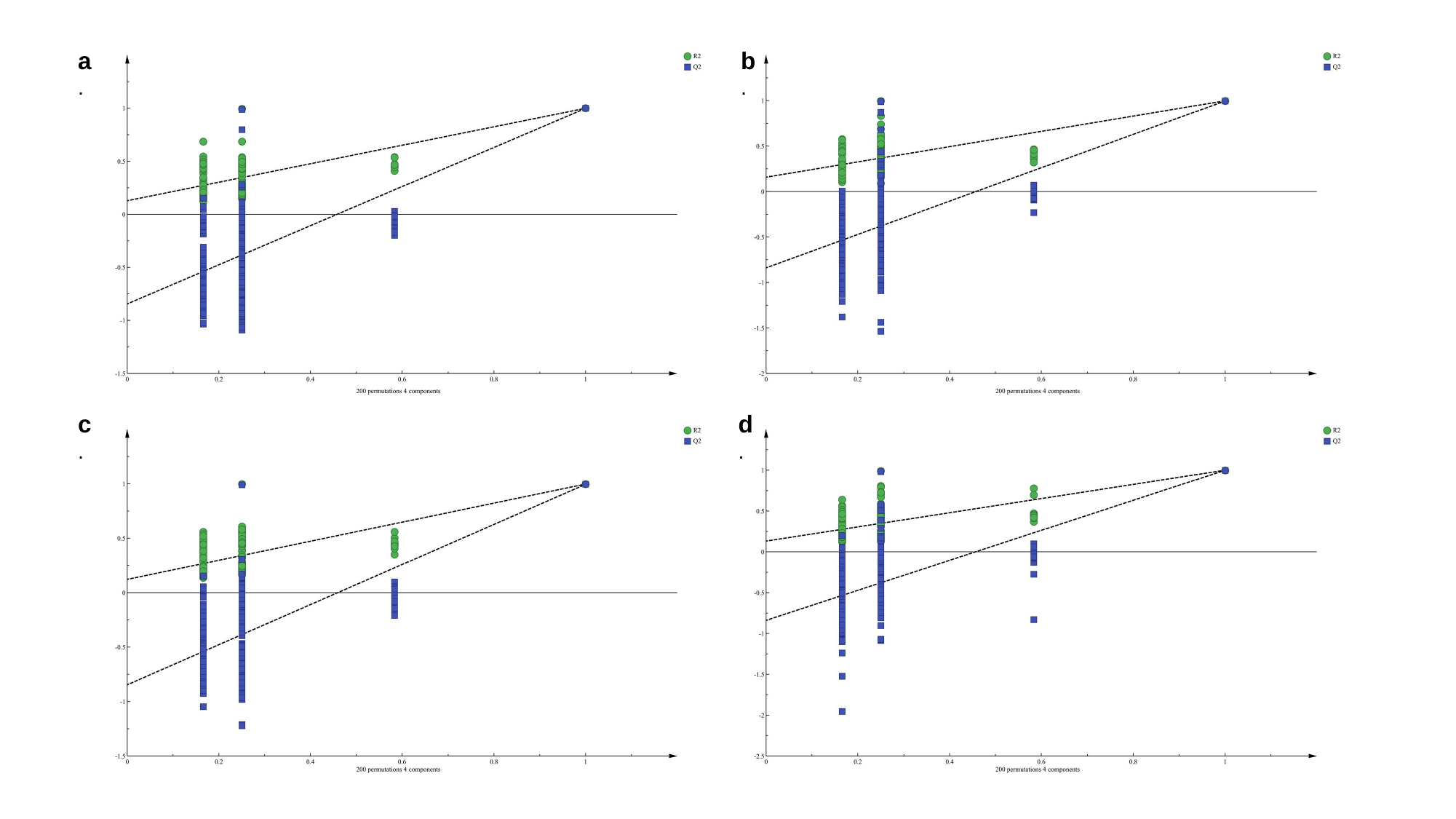

a.
b.
c.
d.

Supplement: Supplementary file 1 [file foods-12-02730-s001.zip › Supplementary Figure.pptx]
